# Test positivity and clinical presentation of COVID-19 in Mozambican infants hospitalized during the second wave of the pandemic in 2021

**DOI:** 10.11604/pamj.2023.44.154.38926

**Published:** 2023-03-30

**Authors:** Dulce Osório, Suraia Tane Liasse, Uneisse Cassia, Muhammad Sidat, Sérgio Taunde, Belarmina Mate, Elcídio Pambo, Otília Mazivila, Beatriz Elias, Cesaltina Lorenzoni, Chris Buck

**Affiliations:** 1 Hospital Central de Maputo, Maputo, Mozambique,; 2 Universidade Eduardo Mondlane School of Medicine, Maputo, Mozambique,; 3 University of California Los Angeles David Geffen School of Medicine, Maputo, Mozambique

**Keywords:** COVID-19, SARS-CoV-2, infant, hospitalization, Mozambique

## Abstract

**Introduction:**

during the second wave of the COVID-19 pandemic in Mozambique, there was a surge in pediatric hospitalizations at a time when there was relatively little evidence, but significant concern about clinical outcomes in African children, particularly in higher-risk infants requiring, and health system capacity to respond.

**Methods:**

a retrospective cohort study was conducted for patients 1-12 months of age admitted to the Breastfeeding ward at Hospital Central de Maputo from January-February 2021. All had routine SARS-CoV-2 PCR testing performed. For patients with positive results, hospital charts were retrospectively reviewed. Descriptive analyses were performed.

**Results:**

of 209 patients that had SARS-CoV-2 PCR testing performed, 102 (48.8%) received results, of which 37 (36.3%) were positive. Positive results were received prior to discharge for 14 patients (37.8%). Median duration of hospitalization was 3 days. There were two deaths in COVID-positive patients (5.4%), both with complex comorbidities. For the 35 COVID-19 positive patients whose charts were located, the principal admission diagnosis was respiratory for 22 (62.9%), and 14 (40.0%) had oxygen saturation <94% at admission. The white blood cell count was >12.0 x 10^3^cells/mL in 10 patients (28.6%) and the most common abnormal finding on chest radiograph was peribronchial thickening (38.5% of patients with results). Oxygen therapy was needed for 20 patients (57.1%).

**Conclusion:**

the majority of infants with COVID-19 had a mild, short-duration respiratory illness that did not exceed ward capacity for care, including oxygen treatment. Laboratory capacity for PCR testing was overwhelmed, delaying the return of results and complicating inpatient infection control measures.

## Introduction

The emergence of the novel SARS-CoV-2 virus and associated COVID-19 disease prompted the World Health Organization (WHO) to declare a public health emergency of international importance in January 2020, with subsequent classification as a global pandemic in March 2020 [1]. The first wave appeared later, and progressed slower in Africa than in other parts of the world, with most countries in sub-Saharan Africa experiencing a larger second wave of disease in late 2020 and early 2021 [2]. In Mozambique, the first wave of cases occurred from August through October 2020, and the second wave began in January 2021, with a median number of daily cases diagnosed four times higher than the first wave, and a much higher number of deaths [3]. The national vaccination program began in March 2021, near the end of the second wave. At the time of the onset of the second wave in Mozambique, very little was known about the clinical presentation of COVID-19 disease in African children, with only two published studies of relatively small cohorts identified on initial literature review. One was a retrospective cohort study from Nigeria that included children identified through community contact testing with a mean age of 12.63 years with only 3.8% of patients in the 0-4 year age group; and the other was a retrospective cohort study from Guinea that included hospitalized children with a mean age of 9.66 years [4,5]. The lack of data from African infants was of concern given early evidence that young age was a significant risk factor for more severe COVID-19 disease [6,7].

Systematic reviews and meta-analyses of studies, primarily from Asia, Europe, and North America had reported that children were more likely to be asymptomatic than adults, and when symptomatic, were less likely to have severe disease [8-12]. The most frequent symptoms in children were fever, rhinorrhea, and cough, often with a clinical picture resembling viral bronchiolitis [13]. There were also reports of a multisystem inflammatory condition that resembled Kawasaki disease in children with SARS-CoV-2 infection [8,14-16]. In infants there were also reports of SARS-CoV-2 infection presenting as fever without a source without respiratory symptoms, but occasionally with neurologic symptoms that prompted lumbar punctures to evaluate for possible meningitis [17,18]. As the second wave unfolded in Mozambique, there was a high level of concern and uncertainty about the impact of COVID-19 on African children, and in particular high-risk infants, who often have other underlying comorbidities including HIV and malnutrition, and the capacity of the resource-limited healthcare systems to be able to respond to the surging pandemic particularly in terms of testing and treatment [19-21]. In this context, this study was conducted to help address some of these knowledge gaps by characterizing inpatient SARS-CoV-2 testing results, clinical characteristics, and outcomes of hospitalized infants diagnosed with COVID-19.

## Methods

**Setting**: the study was conducted on the Breastfeeding Ward at Hospital Central de Maputo (HCM), the largest academic and referral hospital in Mozambique. The ward has a capacity of 44 beds, and admits patients aged 1-12 months with various disease pathologies with approximately 1000 admissions per year. The ward has centralized oxygen which can be delivered up to 10L/min flow, but does not have continuous positive airway pressure or mechanical ventilation capacity. During the time period of this study, HCM policy was for all admitted patients to be tested for SARS-CoV-2 by polymerase chain reaction (PCR) in a centralized national lab, with a turn-around time of approximately 5-7 days. Rapid antigen tests were not routinely used during this time period. With the exception of patients requiring specialized intensive or surgical care, policy was to transfer positive patients needing ongoing hospital care to a designated local COVID-19 treatment facility.

**Design and participants**: this was a retrospective case series study of infants who were discharged from the Breastfeeding Ward at HCM during the second wave of the COVID-19 pandemic in Mozambique, between January 1 to February 28, 2021. The ward register was used to identify eligible patients 1-12 months of age with positive SARS-CoV-2 PCR test results, including those patients whose results returned after discharge. Patients who did not have SARS-CoV-2 PCR testing performed or for whom results were unavailable were excluded.

**Data collection and analysis**: hospital charts were retrieved from the archives for patients with positive test results identified in the ward discharge register. Demographic, clinical, and laboratory data were extracted using paper forms, with subsequent data entry into a Microsoft Excel® database. For patients with chest radiographs performed during hospitalization, images were interpreted using a standardized approach by a single reviewer. Frequencies and medians with interquartile ranges were used to characterize the ward positivity rate and the clinical, laboratorial, and radiological presentation of COVID-19 in the cohort. No imputation was performed for missing data. Microsoft Excel® was used for all analyses.

**Ethical considerations**: the study was conducted according to the guidelines of the Declaration of Helsinki, and had approval from the Institutional Review Board of the Universidade Eduardo Mondlane School of Medicine/Hospital Central de Maputo (CIBS FM&HCM/P030/2020). No informed consent was needed for the secondary analysis of routine clinical data, and the study database included no personal identifiers.

## Results

**Figure 1:**
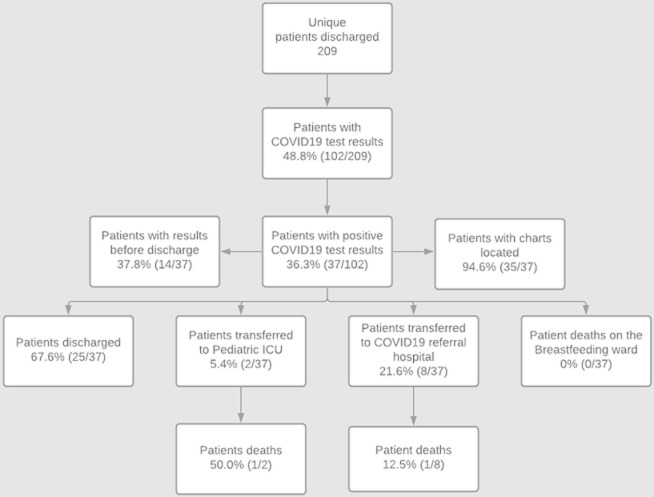
study inclusion, Breastfeeding Ward, Maputo Central Hospital, January-February 2021

**Testing**: there was a total of 209 unique discharges from the ward during the time period of the study. Two patients who were readmitted to the Breastfeeding Ward after initial transfer to the local COVID-19 treatment hospital were only counted once. In the 102 patients with SARS-CoV-2 PCR results (48.8% of all discharges), 37 patients were positive (36.3%). PCR results were received before discharge for 14 patients (37.8%), Figure 1.

**Hospitalization outcomes**: of the 37 patients with COVID-19, 25 (67.6%) were discharged home directly from the Breastfeeding Ward, 2 (5.4%) were transferred to the pediatric intensive care unit (PICU) at HCM, and 8 (21.6%) were transferred to the local COVID-19 referral hospital. The median duration of hospitalization on the Breastfeeding ward prior to discharge or transfer was 3 days (IQR: 2-5), with 19 patients (51.4%) having hospitalizations of <3 days. There were 2 patients (5.4%) that were transferred and died during active COVID-19 disease, both HIV-negative but with complex underlying conditions, namely cerebral atrophy with developmental delay and hepatobiliary cyst, Figure 1. There was an additional death that occurred in an HIV-exposed, uninfected patient with hydrocephalus who had already returned to HCM from the COVID-19 referral hospital after treatment and negative testing that was deemed not related to COVID-19.

**Demographic and historical characteristics**: charts were located for 35 (94.6%) of patients who tested positive. The median age was 4 months (IQR: 2-7), and 23 (65.7%) were male. There was a history of an outpatient visit for the same illness prior to hospitalization in 14 (40.0%) of patients, and 12 (34.3%) of patients were transferred from another health facility for admission. Only 2 (5.7%) had a known COVID-19 contact.

**Clinical characteristics**: the principal admission diagnosis was respiratory in 22 patients (62.9%), specifically bronchiolitis and pneumonia/bronchopneumonia in 17 (48.6%) and 5 (17.1%) of patients, respectively. There was a significant underlying chronic comorbidity in 12 patients (34.3%), specifically hydrocephalus (5 patients), HIV (2 patients), congenital heart disease (1 patient), hepatobiliary cyst (1 patient), velopharyngeal insufficiency (1 patient), cerebral atrophy/severe developmental delay (1 patient), and severe malnutrition (1 patient). Respiratory symptoms were reported in 30 patients (85.7%) with cough, increased work of breathing, and rhinorrhea being the three mostly common symptoms, reported in 29 (82.9%), 28 (80.0%), and 25 (71.4%) of patients, respectively. On admission exam, 11 patients (31.4%) were tachypneic and 8 (22.9%) had oxygen saturation <90% on room air. There was a history of fever in 24 (68.6%) patients, but only 8 (22.9%) were febrile at initial presentation. An abnormal admission lung auscultation exam was documented in 28 patients (80.0%) with rhonchi, wheezing, and crackles reported in 25 (71.4%), 17 (48.6%), and 15 (42.9%) of patients, respectively. Gastrointestinal complaints were reported in a total of 10 patients (28.6%), Table 1.

**Table 1:** clinical characteristics at admission for infants hospitalized with COVID-19 at Hospital Central de Maputo, 2021

Variables	Total 100% (35/35)
**Principal admission diagnosis**	
Bronchiolitis	48.6% (17/35)
Pneumonia/bronchopneumonia	14.3%(5/35)
Hydrocephalus	14.3% (5/35)
Malaria	8.6% (3/35)
Cervical adenitis	5.7% (2/35)
Gastroenteritis	5.7% (2/35)
Abdominal mass	2.9% (1/35)
**Significant underlying comorbidity**	34.3%(12/35)
**Reported symptoms**	
Cough	82.9% (29/35)
Increased work of breathing	80.0%(28/35)
Rhinorrhea	71.4% (25/35)
Fever	68.6% (24/35)
Decreased oral intake	42.9% (15/35)
Diarrhea	25.7% (9/35)
Vomiting	20.0% (7/35)
**Temperature**	
>38.0° C	22.9% (8/35)
37.5-37.9° C	17.1% (6/35)
<37.4° C	60.0% (21/35)
**Respiratory rate**	
>50 breaths per minute	31.4% (11/35)
<50 breaths per minute	68.6% (24/35)
**Oxygen saturation (on room air)**	
95-100%	60.0% (21/35)
90-94%	17.1% (6/35)
85-89%	20.0%(7/35)
80-84%	2.9% (1/35)
**Lung examination**	
Rhonchi	71.4% (25/35)
Wheezing	48.6% (17/35)
Crackles	42.9% (15/35)
**Acute nutritional status**	
No acute malnutrition	60.0% (21/35)
Mild acute malnutrition	14.3% (5/35)
Moderate acute malnutrition	11.4% (4/35)
Severeacute malnutrition	5.7% (2/35)
Missing data	8.6% (3/35)

**Laboratory profile**: all 35 patients had HIV testing and full blood count results from admission. Two patients (5.7%) were HIV-positive and 7 (20%) were HIV-exposed/uninfected. The median white blood cell count for all patients was 10.6 x 10^3^ cells/mL (IQR: 7.5-13.7), with 10 patients (28.6%) having an elevated white blood cell counts (defined as >12.0 x 10^3^ cells/mL). The median absolute neutrophil and lymphocyte counts for all patients were, 4.1 x 10^3^ cells/mL (IQR: 2.7-6.0) and 5.0 x 103 cells/mL (IQR: 3.5-7.5), respectively. Moderate anemia (defined as hemoglobin 8.0-11.9 g/dL) was noted in 29 patients (82.9%) and severe anemia (defined as hemoglobin <7.9 g/dL) in 3 (8.6%), with a median hemoglobin of 10.3 g/dL (IQR: 9.3-11.1). The median platelet count was 417 x 10^3^/mL (IQR: 327-547), with 13 patients (37.1%) having platelet count >500 x 10^3^/mL. Of the 17 patients (48.6%) with alanine aminotransferase (ALT) performed, the median result was 18.8 U/L (IQR: 9.5-22.9), and 3 patients (17.6%) had elevated results. And of the 24 patients (68.6%) with serum creatinine results, 1 (4.2%) had elevated results for age. Further disaggregated laboratory results are presented in Table 2.

**Table 2:** laboratory results at admission for infants hospitalized with COVID-19 at Hospital Central de Maputo, 2021

Variables	Total 100% (35/35)
**HIV**	100% (35/35)
Positive	5.7% (2/35)
Exposed/uninfected	20.0% (7/35)
Negative	74.3% (26/35)
**Full blood count**	
White blood cells (X 103/mL)	100%(35/35)
<4.9	8.6%(3/35)
5.0-11.9	62.9% (22/35)
12.0-17.9	8.6% (3/35)
>18.0	20.0%(7/35)
Neutrophils (X 103/mL)	91.4% (32/35)
<500	0% (0/32)
500-999	3.1% (1/32)
1000-4999	59.4% (19/32)
5000-9999	21.9% (7/32)
>10,000	15.6% (5/32)
Lymphocytes (X 103/mL)	94.3% (33/35)
<500	0%(0/33)
500-999	3.0% (1/33)
1000-4999	45.5%(15/33)
5000-9999	48.5%(16/33)
>10,000	3.0% (1/33)
Hemoglobin (g/dL)	100%(35/35)
<7.9	8.6% (3/35)
8.0-11.9	82.9% (29/35)
>12	8.6% (3/35)
Platelets (X 103/mL)	100% (35/35)
<149	2.9%(1/35)
150-350	28.6%(10/35)
351-499	31.4%(11/35)
>500	37.1%(13/35)
**Biochemistry**	
ALT results available	17/35 (48.6%)
Normal	82.4% (14/17)
1-2.9 times ULN	17.6% (3/17)
Creatinine results available	68.6%(24/35)
Normal	95.8%(23/24)
1-2 times ULN	0%(0/24)
>3 times ULN	4.2%(1/24)

**Chest radiography characteristics**: of the 26 (74.3%) patients with an admission chest radiograph, 14 (53.8%) had at least one abnormal finding. The most frequent abnormal findings were peribronchial thickening and low-density peripheral opacities, seen in 10 (38.5%) and 3 (11.5%) of chest radiographs, Table 3.

**Table 3:** chest radiography findings at admission for infants hospitalized with COVID-19 at Hospital Central de Maputo, 2021

Variables	Total 100% (35/35)
**Chest radiography performed**	74.3%(26/35)
**Findings**	
No abnormal findings	46.2% (12/26)
Peribronchial thickening	38.5% (10/26)
Low-density peripheral opacities	11.5% (3/26)
Lobar pneumonia	3.8% (1/26)
Atelectasis	3.8% (1/26)

**Inpatient treatment**: a total of 20 patients (57.1%) required oxygen support via nasal cannula or face mask. In the 14 patients who were diagnosed with COVID-19 prior to hospital discharge, 11 (78.6%) received antibiotics, 4 (28.6%) received corticosteroids, and 4 (28.6%) received bronchodilators. In the 21 patients who were only diagnosed with COVID-19 after discharge, 8 (38.1%) received antibiotics, 8 (38.1%) received corticosteroids, and 7 (33.3%) received bronchodilators during hospitalization, Table 4.

**Table 4:** inpatient treatment summary for infants hospitalized with COVID-19 at Hospital Central de Maputo, 2021

Variables	COVID-19 diagnosis before discharge	COVID-19 diagnosis after discharge	Total
**Number of patients**	40.0% (14/35)	60.0% (21/35)	100% (35/35)
**Required oxygen support**			
Nasal cannula/face mask	57.1% (8/14)	57.1% (12/21)	57.1% (20/35)
None	42.9% (6/14)	42.9% (9/21)	42.9% (15/35)
**Antibiotics**			
Yes	78.6%(11/14)	38.1% (8/21)	54.3% (19/35)
No	21.4% (3/14)	61.9% (13/21)	45.7% (16/35)
**Corticosteroids**			
Yes	28.6%(4/14)	38.1%(8/21)	34.3% (12/35)
No	71.4% (10/14)	61.9% (13/21)	65.7% (23/35)
**Bronchodilators**			
Yes	28.6% (4/14)	33.3% (7/21)	31.4%(11/35)
No	71.4% (10/14)	66.7% (14/21)	68.6% (24/35)

## Discussion

This study assessed the prevalence of SARS-CoV-2 infection in infants hospitalized at HCM during the second wave of the COVID-19 pandemic in early 2021, and found that over one third (36.3%) of infants with available PCR test results were positive. We were not able to identify any other studies from sub-Saharan Africa that reported on the prevalence of COVID-19 in hospitalized children, but a study from Cape Town, South Africa reported a hospitalization rate of 39% in children who tested positive, with a higher risk for hospitalization in infants <12 months of age [22]. Another study from Tshwane District in Gauteng Province South Africa showed that pediatric admissions typically surged with increases in testing and test positivity, with patients <1 year representing 35% of pediatric admissions during the fourth wave there [23]. It seems clear that during COVID-19 waves, the burden of disease in hospitalized African children, particularly infants, will be greater.

The mortality rate for infants with COVID-19 was 5.4%, which is slightly higher than reported mortality rates of 3-4% from published studies from South Africa that included older children, but still consistent with results from a sub-Saharan Africa analysis that showed relatively low death rates in children and infants [22-24]. A recent report from 25 hospitals in 6 African countries identified young age as a significant risk factor for mortality which might help explain the slightly higher mortality rate from this study that only included infants <12 months of age [25]. Both of the patients who died in this study had complex underlying chronic illness, and these findings align with other reports showing that COVID-19 infection is more often a contributing factor to mortality in children with preexisting comorbidities rather than a principal cause of death in otherwise healthy children [23,25,26]. The clinical picture of the majority of the infants with COVID-19 in this study was similar to that of bronchiolitis with 68.6% of patients having a history of fever, 85.7% having at least one reported respiratory symptom on admission history, with a mix of rhonchi, wheezing, and crackles noted on the admission lung exam. However, 31.5% of patients did not have a primary respiratory diagnosis, similar to the study from Tshwane, South Africa that reported 36% of pediatric patients had an incidental COVID diagnosis while hospitalized for other reasons [23]. The severity of disease assessed by the proportion of admitted patients who needed some type of respiratory support was lower in this cohort of Mozambicans infants compared to infants from the Cape Town South African cohort (57.1% vs. 73.9%), and similarly, the proportion of infants needing intensive care was lower in the Mozambican cohort of infants (5.4% vs. 21.7%) [22]. It should be noted that some patients in this cohort required oxygen therapy for increased work of breathing and not for hypoxemia.

During the height of the pandemic, there was much concern that patients with tuberculosis could have a more severe clinical presentation of acute COVID-19 infection, or experience diagnostic delays due to common presenting symptoms. We did not have any patients in our COVID-19 positive cohort with preexisting or new TB diagnoses during hospitalization [27]. Nor did we have any patients with a multisystem inflammatory syndrome, and published reports from South Africa and Nigeria have shown that this presentation is more common in older children than in infants [28-30]. And diarrhea may be more frequent in infants as it was noted in 25.7% of the patients in our cohort, but was reported in 18.4% of all age children from 8 African countries in a systematic review [31].

The leukocyte profile from full blood count testing of infants with COVID-19 in this study was similar to what was reported from the cohort of hospitalized children of all ages from Tshwane, South Africa with the majority of Mozambican infants having a normal absolute white blood cell count, and rare neutropenia or lymphopenia. One notable difference was a lower median hemoglobin in the Mozambican cohort compared to the South African children (10.3 vs. 12.5 g/dL) which likely reflects overall health and nutrition prior to admission [23]. With respect to chest radiograph findings, a much higher percentage of Mozambican infants had normal exams compared to the cohort of infants from Cape Town, South African (46.2% vs. 6%) [22].

One operational finding that was particularly worrisome from this study was that we received SARS-CoV-2 PCR results for fewer than half of patients (48.8%) who were admitted during the time period of the study, despite universal sample collection. And only 37.8% of patients with COVID-19 received their PCR results during their hospitalization. The national central laboratory capacity for timely PCR testing was insufficient to meet demands with gaps in specimen collection, transportation, processing, and results delivery, similar to what was seen in other African settings in the early waves of the pandemic [32-34]. This undoubtedly contributed to nosocomial transmission to other patients and healthcare workers as we were unable to set up any type of effective inpatient isolation strategy without test results for patients or their accompanying caregivers. Furthermore, without test results, clinicians were unable to initiate COVID-19 treatment per national guidelines. We found that, for example, patients who were diagnosed after discharge were much less likely to have received antibiotics during hospitalization despite their being recommended in the national pediatric COVID-19 treatment algorithm in place at the time of this study. Fortunately, the Pediatrics Department at HCM introduced rapid antigen-based testing as a first screening test for patients requiring hospitalization later in 2021 while the Ministry of Health worked to expand PCR capacity, with dramatic improvements noted in our capacity to isolate and refer patients to designated COVID-19 referral hospitals when needed [3].

This study was unique in that provides a detailed characterization of the clinical, laboratory, and radiologic presentation of COVID-19 in African infants, a population that has not been well-described in the literature with respect to COVID-19 infection. But the study had several limitations which also need mention including the relatively small sample size from a single site, the low return rate of SARS-CoV-2 PCR tests which likely precluded the inclusion of other positive infants, suboptimal quality of some printed chest radiographs, and the lack of a comparison group of hospitalized COVID-19-negative infants due to the manual data collection method used. It must also be noted that during the time period of this study, the predominant type of SARS-CoV-2 in Mozambique was the Beta variant [3]. Evidence from other countries have shown differences in the severity and presentation of infection with other variants such as Delta and Omicron and similar studies could be performed for subsequent waves for comparison [23,35].

## Conclusion

In the second wave of the pandemic in Mozambique, the majority of infants hospitalized with COVID-19 had a mild, short-duration bronchiolitis-type respiratory illness that did not exceed ward capacity for care, including oxygen treatment. But laboratory capacity for PCR testing was overwhelmed, and initial screening with rapid diagnostic tests is essential for timely case identification and inpatient infection control measures. These results are pertinent for other hospitals in Mozambique and the region, and can help inform plans for the hospital care of infants in possible future waves of COVID-19.

### What is known about this topic

- *COVID-19 has been reported to have a varied clinical presentation in infants including asymptomatic, fever without a source, respiratory disease, and multisystem inflammatory syndrome;*
- *Most children tend to have mild disease, but younger age and underlying comorbidities are significant risk factors for more severe COVID-19 disease and mortality;*
- *The laboratory capacity for COVID-19 diagnosis was a major challenge for many sub-Saharan African countries in the early phases of the pandemic*.

### What this study adds

- *The majority of Mozambican hospitalized infants with COVID-19 during the second wave of the pandemic, when the Beta strain predominated, had a clinical, radiological, and laboratory presentation consistent with a mild bronchiolitis-type illness; there were no cases of suspected or confirmed multisystem inflammatory syndrome;*
- *The mortality rate in this cohort of hospitalized infants was 5.4%, and both of the infants who died had complex underlying illness;*
- *The capacity for laboratory diagnosis of COVID-19, which depended on PCR testing at the time of the study, was overwhelmed with delays in results delivery and missing results, complicating efforts to isolate cases and implement effective inpatient infection control measures*.
